# Associations of Visceral Adiposity Index and Body Roundness Index with chronic kidney disease in a Hakka population: mediating effect of blood pressure

**DOI:** 10.3389/fnut.2026.1732017

**Published:** 2026-02-05

**Authors:** Jinxia Su, Shengzhu Huang, Binran Zhao, Qiuyan Tan, Shanshan Li, Jing Huang, Boning Hu, Xiaolai Li, Bijun Li, Chen Li, Zengnan Mo, Ling Pan, Wei Li

**Affiliations:** 1 Department of Nephrology, The Second Affiliated Hospital of Guangxi Medical University, Guangxi Medical University, Nanning, Guangxi, China; 2 Center for Genomic and Personalized Medicine, Guangxi Key Laboratory for Genomic and Personalized Medicine, Guangxi Collaborative Innovation Center for Genomic and Personalized Medicine, Guangxi Medical University, Nanning, Guangxi, China; 3 Bobai County People’s Hospital, Yulin, Guangxi, China; 4 Institute of Urology and Nephrology, The First Affiliated Hospital of Guangxi Medical University, Guangxi Medical University, Nanning, Guangxi, China; 5 Department of Nephrology, The First Affiliated Hospital of Guangxi Medical University, Guangxi Medical University, Nanning, Guangxi, China

**Keywords:** association, Body Roundness Index, chronic kidney disease, mediation effect, Visceral Adiposity Index

## Abstract

**Objective:**

This study aims to investigate the prevalence of chronic kidney disease (CKD) and explore the associations of the Visceral Adiposity Index (VAI) and Body Roundness Index (BRI) with CKD risk in the Hakka population, while quantitatively assessing the mediating effects of blood pressure indicators.

**Methods:**

Data for this study were obtained from a cross-sectional survey conducted in Bobai County, Guangxi, which included 8971 adult participants. Log-binomial regression, robust Poisson regression, and restricted cubic spline regression were used to evaluate the associations between the VAI/BRI and CKD risk. Mediation analysis was further conducted to assess the role of blood pressure parameters in these associations.

**Results:**

The overall prevalence of CKD was 12.62% in the study population. In the fully adjusted multivariable model (Model 3), compared with the lowest quartiles (Q1, reference), the highest quartiles (Q4) of both VAI and BRI were significantly associated with increased risks of CKD, with prevalence ratios of 1.61 (95% CI: 1.32–1.98) and 1.26 (95% CI: 1.05–1.52), respectively. Restricted cubic spline analysis revealed nonlinear associations between VAI/BRI and CKD risk, with accelerated risk increments at higher levels of both indices. Mediation analysis revealed that blood pressure indicators significantly mediated the VAI/BRI-CKD relationships, with notably stronger effects observed for BRI (SBP: 42.69%; DBP: 51.16%; MAP: 53.38%) compared to VAI (SBP: 21.4%; DBP: 28.15%; MAP: 28.41%). Additionally, PP exhibited a significant mediating effect only in the BRI-CKD pathway (12.21, 95% CI: 7.28–20.70).

**Conclusion:**

The VAI and BRI were independent risk factors for CKD in Hakka Biobank (HKB), with their associations with CKD are partially mediated by blood pressure. Given their ease of measurement and cost-effectiveness, VAI and BRI may serve as practical screening tools for identifying CKD high-risk individuals in community populations, thereby enabling early intervention and targeted prevention strategies of CKD.

## 1 Introduction

Chronic kidney disease (CKD) has emerged as a major global public health challenge, primarily driven by its steadily rising prevalence, high rates of disability and mortality, and strong association with cardiovascular events (1, 2). Concurrently, the global obesity epidemic has intensified markedly over the past two decades (3). Notably the parallel trends in prevalence of CKD and obesity have prompted in-depth investigation into their potential interconnection.

Substantial evidence confirms the obesity as a major risk factor for the development and deterioration of CKD (4). A meta-analysis encompassing over 630,000 participants demonstrated that obesity elevates the risks of albuminuria and renal function decline by 51 and 28%, respectively (5); Furthermore, another meta-analysis further revealed that obese individuals face an 83% higher risk of developing kidney disease compared to those with normal weight (95% CI: 1.57–2.13) (6). The association between obesity and CKD is largely mediated by hypertension: research has shown that obesity as a significant risk factor for hypertension (7, 8), which involves mechanisms such as sympathetic nervous system (SNS) activation, renin-angiotensin-aldosterone system (RAAS) overactivity, and induction of insulin resistance. These alterations not only directly promote the development of hypertension but also lead to glomerular hypertension, hyperfiltration, and microvascular damage, accelerating the onset and progression of CKD (9). More pivotally, systolic blood pressure (SBP) has been demonstrated to exert independent and cumulative adverse effects on both microvascular and macrovascular complications in CKD (10), further establishing the critical mediating role hypertension of in the obesity–CKD relationship.

However, current understanding of the “obesity–hypertension–CKD” pathway remains limited both in practical application and mechanistic insight. On the practical level, direct measurement of visceral adipose tissue (VAT) through gold-standard methods such as computed tomography or magnetic resonance imaging remains costly and technically complex, which significantly limits its applicability for large-scale population screening. Furthermore, traditional anthropometric measurements [e.g., body mass index (BMI), waist circumference (WC)] for detecting visceral obesity remains limited, highlighting the need to incorporate additional parameters to strengthen their practical utility. On the mechanistic level, although existing evidence suggests a mediating role of blood pressure in the VAT–CKD association (11), direct evidence regarding its mediation in relationships involving novel adiposity indices remains scant. These critical limitations have stimulated the development of novel adiposity indices for early identification of individuals at high-risk of CKD, with the Visceral Adiposity Index (VAI) and Body Roundness Index (BRI).

Specifically, VAI is a functional composite measure that integrates WC, BMI, triglycerides (TG), and high-density lipoprotein cholesterol (HDL-C). This multi-parameter integration enables VAI to not only reflect the accumulation of VAT but also accurately to assess the metabolic activity and functional status (12). Evidence indicates that the VAI is an independent risk marker for CKD (13). It also shows potential in predicting CKD pathogenesis and is negatively associated with estimated glomerular filtration rate (eGFR) (14). And BRI is an anthropometric index derived from height and WC measurements. It estimates body fat distribution, has been validated as a reliable surrogate marker of visceral fat accumulation and central obesity (15). BRI is an independent marker of CKD risk in individuals with diabetes and may reflect contributions from glycaemic control, kidney function, and nutritional status (16). These two indicators are also significantly associated with the risk of various diseases, including cardiovascular disease (CVD), hypertension, type 2 diabetes, and metabolic syndrome, exhibiting superior performance in risk prediction compared to traditional anthropometric measures such as BMI and WC (17–21). A key advantage of these two indices lies in their practicality: they can be calculated directly from standard clinical measurements, without necessitating any specialized assessments, making them straightforward to implement and standardize, and making them well-suited for implementation in primary healthcare settings and large-scale epidemiological studies. While the associations of VAI and BRI with CKD have been established in other populations (e.g., the US), evidence from Chinese cohorts, particularly southern Chinese populations, remains scarce. Moreover, the potential mediating role of blood pressure, in the VAI/BRI–CKD association has yet to be quantified.

Therefore, this study aims to address these gaps by leveraging cross-sectional survey data from the Hakka population in Bobai County, Guangxi, which is an ethnic subgroup of the Han ethnicity. Specifically, the study will investigate the prevalence and distribution characteristics of CKD in this population, examine the associations between VAI/BRI and CKD. Furthermore, we will quantitatively assess the mediating effects of blood pressure indicators in these associations and systematically compare potential differences in the mediating pathways between the two adiposity indices. We hope that our research can provide new insights into the treatment and prevention of CKD and offer theoretical support for clinical decision-making.

## 2 Methods

### 2.1 Study population

Data for this study were obtained from a community-based cross-sectional survey conducted in Bobai County, Guangxi, from July 20 to August 10, 2024. The study was implemented in 15 selected communities within the country, and participants were recruited through joint promotion and mobilization by government and community organization. The inclusion criteria were community residents aged ≥18 years with local residency ≥6 months.

**Figure 1:**
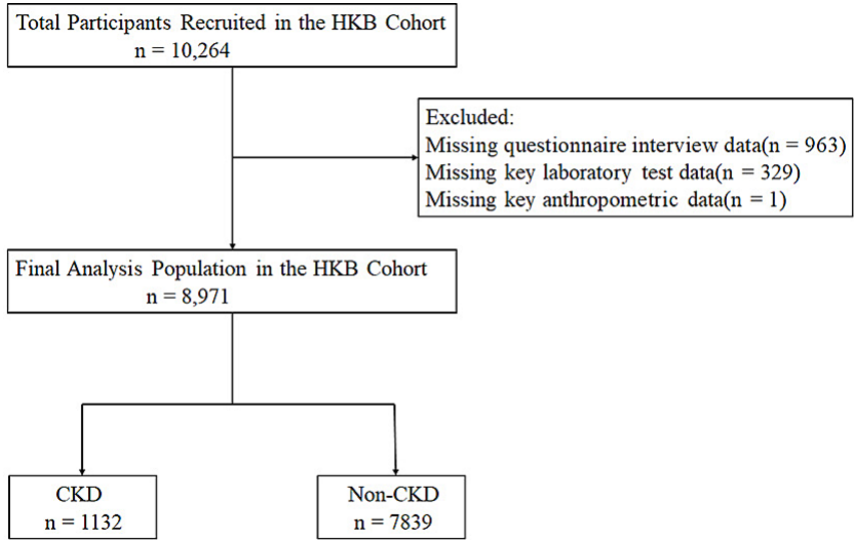
Flow chart of the study participants.

A total of 10,264 were initially enrolled in the study. After data quality verification, 8,971 participants with complete questionnaire information and no missing key physical measurement or laboratory measurements test data were finally retained for the statistical analysis (Figure 1). Among the final analytical population, 6,994 participants (accounting for 78%) were from the Hakka ethnic group. According to the presence or absence of CKD, the study population was further divided into two groups: the CKD group (1,132 participants, 12.62%), the non-CKD group (7,839 participants, 87.38%).

The study protocol was approved by the Ethics Review Committee of Bobai County People’s Hospital (Approval No.: 2024-KY-026). All participants signed a written informed consent form before the start of the study, and the entire research process strictly adhered to the ethical principles of the *Declaration of Helsinki*.

### 2.2 Data collection

Demographic and socioeconomic data, including age, sex, ethnicity, marital status, smoking status, alcohol intake, education level, and medical history, were collected at the time of study enrollment using a structured questionnaire.

Physical examinations were conducted by trained medical staff in accordance with standardized protocols, covering measurements of height, weight, WC, hip and blood pressure.

For the laboratory assessment, all participants fasted for at least 10 h prior to sample collection. Blood and urine specimens were collected between 8:00 and 10:00 a.m. Immediately after collection, samples were stored at 2–8 °C and transported to the clinical laboratory of Bobai County People’s Hospital, where serum and plasma separation was completed within 4 h of sample acquisition to ensure sample quality. All laboratory analyses were performed using automated analyzers. Blood measurements included fasting plasma glucose (FPG), total cholesterol (TC), TG, HDL-C, low-density lipoprotein cholesterol (LDL-C), serum creatinine, and blood urea nitrogen. Urine measurements included urinary albumin (ALB) and urinary creatinine (UCR), and the urine albumin-to-creatinine ratio (UACR) was subsequently calculated using these two parameters.

### 2.3 Definitions and formulas

The CKD was defined as an estimated glomerular filtration rate (eGFR) < 60 mL/min/1.73 m^2^ or a UACR ≥ 30 mg/g. The eGFR was calculated using the CKD Epidemiology Collaboration (CKD-EPI) equation (22).

Educational attainment was categorized as “less than high school” and “high school or above.” Marital status was classified as “married” and “other (including unmarried, divorced and widowed).” Smoking status was defined as non-smoker (including never smokers or former smokers) and current smoker (individuals who smoked at least one cigarette per day for more than 6 months). Alcohol consumption status was determined based on drinking frequency in the past year and classified as non-drinker (including never drinkers or former drinkers) and current drinker.

Hypertension was defined as meeting any of the following criteria: SBP ≥ 140 mmHg, diastolic blood pressure (DBP) ≥ 90 mmHg, self-reported physician diagnosis of hypertension, or current use of antihypertensive medication. Diabetes mellitus was diagnosed if any of the following conditions were met: FPG ≥ 126 mg/dL, self-reported previous diagnosis of diabetes, or current use of glucose-lowering agents. Dyslipidemia was identified by any of the following: TC ≥ 6.2 mmol/L, TG ≥ 2.28 mmol/L, LDL-C ≥ 4.1 mm/L, HDL-C < 0.09 mmol/L, self-reported history of dyslipidemia, or current lipid-lowering therapy. Hyperuricemia was defined as serum uric acid levels >420 μmol/L in men or >360 μmol/L in women, or self-reported physician diagnosis of hyperuricemia.

Formulas for the calculated variables were as follows:


$$\mathrm{BMI}=\frac{\text{Weight}(\mathrm{kg})}{\text{Height}{\left(m\right)}^{2}}$$

VAI was calculated by sex-specific formulas:

$$\text{Male}:\mathrm{VAI}=\frac{\mathrm{WC}}{\left(39.68+(1.88\times \mathrm{BMI}\right)}\times (\frac{\mathrm{TG}}{1.03})\times (\frac{1.31}{\mathrm{HDL}-C})$$

$$\text{Female}:\mathrm{VAI}=\frac{\mathrm{WC}}{\left(36.58+(1.89\times \mathrm{BMI}\right)}\times (\frac{\mathrm{TG}}{0.81})\times (\frac{1.52}{\mathrm{HDL}})$$

$$\mathrm{BRI}=364.2-365.5\times \sqrt{1-{\left(\frac{\mathrm{WC}}{2\pi }\right)}^{2}/{\left(0.5\times \text{height}\right)}^{2}}$$

### 2.4 Statistical analysis

All statistical analyses were performed using R software (version 4.5.0). Continuous variables are expressed as the mean ± standard deviation (SD), while categorical variables were expressed as an absolute number (%). For group comparisons: continuous variables were analyzed independent samples using the t-test if they followed normal distribution; for non-normally distributed continuous variables, the Kruskal–Walli’s test was applied. Categorical variables were compared using the chi-squared (χ^2^) test. Covariates adjusted in the multivariable models included sex, age, education level, marital status, alcohol consumption, smoking status, hypertension, diabetes, hyperuricemia, and dyslipidemia. To investigate the association between VAI/BRI levels [stratified into Quartile 1 (lowest), Quartile 2, Quartile 3, and Quartile 4 (highest)] and CKD, log-binomial regression analysis or robust Poisson regression analysis was used. Restricted cubic spline analysis (RCS) was performed to explore the potential non-linear association between VAI/BRI levels and CKD risk, using a smoothing technique. The spline degrees for freedom for the spline were determined based on the lowest Akaike information criterion to optimize model fit. Subgroup analyses according to sex, age (≤65 vs. >65 years), education level, marital status, alcohol consumption, smoking status, hypertension, diabetes, hyperuricemia, and dyslipidemia to assess the consistency of associations across different populations. Interaction effects between VAI/BRI and subgroup variables on CKD risk were formally tested by including interaction terms in the regression models. To further evaluate the mediation effect of pressure indicators (SBP, DBP, pulse pressure [PP], and mean arterial pressure [MAP]) between VAI/BRI and CKD, mediation analysis was performed. This analysis quantified the indirect effect of VAI/BRI on CKD through each blood pressure indicators, using R package “mediation” with 1,000 bootstrap resamples for 95% confidence intervals (Cl) for the mediating effects. A two-tailed *p-v*alue < 0.05 was considered statistically significant for all analyses.

## 3 Results

### 3.1 Baseline characteristics

The baseline characteristics of study participants according to CKD status are shown in Table 1. Compared with participants in non-CKD group, those with CKD demonstrated significantly higher levels of SBP, DBP, PP, MAP, weight, WC, hip, BMI, VAI, and BRI. In terms of laboratory tests, participants in the CKD group showed exhibited higher levels of FPG, TG, Scr, BUN, UA, ALB, and UACR.

**Table 1:** Baseline characteristics of participants.

Variable	All (*N* = 8,971)	Non-CKD (*n* = 7839)	CKD (*N* = 1,132)	*p*
Sex, *n* (%)				<0.001
Male	3,077 (34.3%)	2,620 (33.4%)	457 (40.4%)	
Female	5,894 (65.7%)	5,219 (66.6%)	675 (59.6%)	
Age, *n* (%)				<0.001
≤65	7,362 (82.1%)	6,584 (84.0%)	778 (68.7%)	
>65	1,609 (17.9%)	1,255 (16.0%)	354 (31.3%)	
Nation, *n* (%)				0.794
Han	8,886 (99.1%)	7,762 (99.0%)	1,124 (99.3%)	
Zhuang	62 (0.69%)	56 (0.71%)	6 (0.53%)	
Other	23 (0.26%)	21 (0.27%)	2 (0.18%)	
Hakka, *n* (%)				0.699
No	1977 (22.0%)	1722 (22.0%)	255 (22.5%)	
Yes	6,994 (78.0%)	6,117 (78.0%)	877 (77.5%)	
Education, *n* (%)				<0.001
Less than high school	5,620 (62.6%)	4,854 (61.9%)	766 (67.7%)	
High school or above	3,351 (37.4%)	2,985 (38.1%)	366 (32.3%)	
Marriage, *n* (%)				0.925
Unmarried/Divorced/Widowed	490 (5.46%)	427 (5.45%)	63 (5.57%)	
Married	8,481 (94.5%)	7,412 (94.6%)	1,069 (94.4%)	
Smoke, *n* (%)				0.832
No	7,905 (88.4%)	6,895 (88.4%)	1,010 (88.7%)	
Yes	1,033 (11.6%)	904 (11.6%)	129 (11.3%)	
Drink, *n* (%)				0.119
No	6,836 (76.2%)	5,952 (75.9%)	884 (78.1%)	
Yes	2,135 (23.8%)	1887 (24.1%)	248 (21.9%)	
Exercise per week, *n* (%)	3.02 (1.73)	3.00 (1.72)	3.16 (1.75)	0.005
SBP, mmHg	134 (21.5)	132 (20.5)	148 (23.6)	<0.001
DBP, mmHg	85.5 (12.0)	84.6 (11.5)	92.3 (13.5)	<0.001
PP, mmHg	48.3 (15.3)	47.2 (14.7)	55.6 (17.5)	<0.001
MAP, mmHg	102 (14.1)	100 (13.4)	111 (15.4)	<0.001
Height, cm	158 (7.52)	158 (7.47)	158 (7.89)	0.934
Weight, kg	61.7 (10.5)	61.5 (10.3)	63.4 (11.7)	<0.001
BMI, kg/m^2^	24.6 (3.36)	24.5 (3.30)	25.3 (3.65)	<0.001
Waist, cm	84.7 (9.88)	84.3 (9.71)	87.8 (10.5)	<0.001
Hip, cm	95.0 (8.08)	94.8 (8.02)	96.0 (8.41)	<0.001
CVD, *n* (%)				<0.001
No	8,715 (97.1%)	7,645 (97.5%)	1,070 (94.5%)	
Yes	256 (2.85%)	194 (2.47%)	62 (5.48%)	
Hypertension, *n* (%)				<0.001
No	4,429 (49.4%)	4,182 (53.3%)	247 (21.8%)	
Yes	4,542 (50.6%)	3,657 (46.7%)	885 (78.2%)	
Antihypertensive medications, *n* (%)				<0.001
No	7,588 (84.6%)	6,852 (87.4%)	736 (65.0%)	
Yes	1,383 (15.4%)	987 (12.6%)	396 (35.0%)	
Diabetes, *n* (%)				<0.001
No	8,015 (89.3%)	7,156 (91.3%)	859 (75.9%)	
Yes	956 (10.7%)	683 (8.71%)	273 (24.1%)	
Diabetes-drugs, *n* (%)				<0.001
No	8,504 (94.8%)	7,516 (95.9%)	988 (87.3%)	
Yes	467 (5.21%)	323 (4.12%)	144 (12.7%)	
Hyperlipidemia, *n* (%)				<0.001
No	2,830 (31.5%)	2,548 (32.5%)	282 (24.9%)	
Yes	6,141 (68.5%)	5,291 (67.5%)	850 (75.1%)	
Lipid-lowering medications, *n* (%)				<0.001
No	8,673 (96.7%)	7,599 (96.9%)	1,074 (94.9%)	
Yes	298 (3.32%)	240 (3.06%)	58 (5.12%)	
Hyperuricemia, *n* (%)				<0.001
No	6,179 (68.9%)	5,557 (70.9%)	622 (54.9%)	
Yes	2,792 (31.1%)	2,282 (29.1%)	510 (45.1%)	
FBG, mmol/L	5.46 (1.50)	5.36 (1.30)	6.16 (2.38)	<0.001
TG, mmol/L	1.93 (1.79)	1.86 (1.66)	2.43 (2.48)	<0.001
TC, mmol/L	5.15 (1.03)	5.14 (1.00)	5.24 (1.22)	0.006
HDL.C, mmol/L	1.25 (0.27)	1.26 (0.27)	1.19 (0.27)	<0.001
LDL.C, mmol/L	3.15 (0.79)	3.14 (0.78)	3.18 (0.84)	0.157
Cr, μmol/L	64.2 (22.0)	62.0 (14.0)	79.0 (47.1)	<0.001
BUN, mmol/L	4.82 (1.35)	4.72 (1.19)	5.56 (2.01)	<0.001
UA, μmol/L	347 (89.0)	342 (86.5)	379 (99.2)	<0.001
ALB, g/L	44.0 (218)	16.5 (10.5)	234 (577)	<0.001
UCR, mmol/L	16.0 (5.27)	16.2 (5.18)	14.7 (5.67)	<0.001
eGFR, mL/min/1.73 m^2^	101 (14.8)	103 (12.0)	88.6 (23.5)	<0.001
UACR	28.5 (165)	9.63 (5.89)	159 (444)	<0.001
VAI	3.05 (5.88)	2.88 (4.96)	4.27 (10.1)	<0.001
BRI	4.13 (1.24)	4.07 (1.21)	4.53 (1.34)	<0.001

SBP, systolic blood pressure; DBP, diastolic blood pressure; PP, pulse pressure; MAP, mean arterial pressure; BMI, body mass index; FPG, fasting plasma glucose; TG, triglyceride; HDL-C, high-density lipoprotein cholesterol; Scr, serum creatinine; BUN, blood urea nitrogen; UA, uric acid; ACR, albumin-to-creatinine ratio; UCR, urinary creatinine; ALB, urine albumin; UACR, urinary albumin-to-creatinine ratio; eGFR, estimated glomerular filtration rate; VAI, visceral adiposity index; BRI, body roundness index; CVD, cardiovascular disease.

Conversely, they had significantly lower levels of HDL-C and eGFR compared to the non-CKD group. Additionally, the prevalence of comorbidities CVD, hypertension, diabetes, and dyslipidemia was significantly higher in the CKD group than in the non-CKD group. All between-group differences mentioned above were statistically significant (All *p* < 0.05).

### 3.2 CKD prevalence

Table 2 presents the prevalence of CKD based on renal function status and albuminuria levels. The overall prevalence of CKD was 12.62% (95% CI: 11.94–13.33%). Among the total CKD participants, the majority were classified based on UACR ≥30 accounted for 11.75%, while eGFR <60 contributed 2.03%.

**Table 2:** Prevalence of CKD by renal function impairment and albuminuria status.

eGFR, ml/min/1.73 m^2^	UACR, mg/g (95%CI)			Total
	<30	30–299	≥300	
≥90	NA	6.84(6.33–7.39)	0.59(0.45–0.78)	7.44(6.90–8.00)
60–89	NA	2.82(2.49–3.19)	0.32(0.22–0.47)	3.44(2.80–3.53)
30–59	0.87(0.69–1.09)	0.69(0.53–0.89)	0.23(0.15–0.36)	1.79(1.53–2.10)
15–29	NA	0.10(0.05–0.20)	0.10(0.05–0.20)	0.20(0.12–0.32)
<15	NA	NA	0.04(0.01–0.12)	0.04(0.01–0.12)
Total	0.87(0.69–1.09)	10.46(9.83–11.11)	1.29(1.07–1.55)	12.62(11.94–13.33)

### 3.3 Association between VAI/BRI and CKD

**Figure 2:**
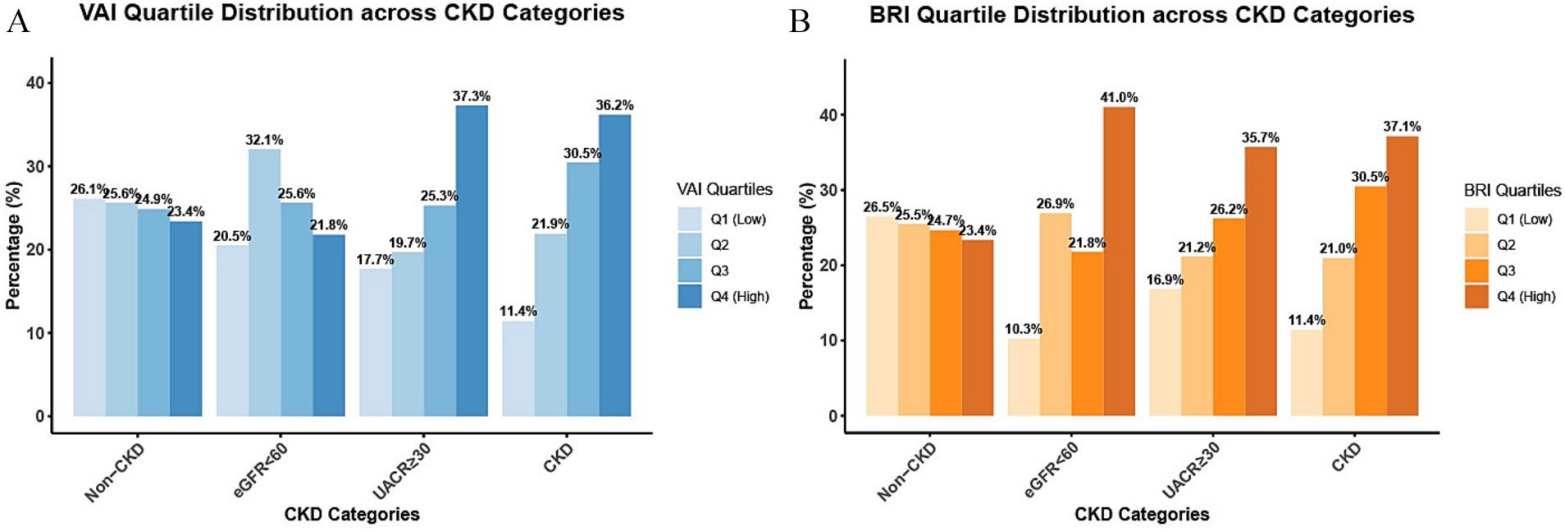
Distribution of VAI/BRI quartiles by renal damage pattern. The Figure shows the distribution proportions of participants across quartiles (Q1–Q4) of VAI **(A)** and BRI **(B)** in the non-CKD group, the eGFR<60group, the UACR≥30 group, and the CKD group.

The distribution of VAI and BRI quartiles revealed distinct patterns across different renal function categories (Figure 2). Specifically, in the UACR ≥30 group, VAI Q4 values accounted for 37.3% and BRI Q4 accounted for 35.7%. Similarly, the CKD group exhibited elevated rates of Q4 values, with 36.2% for VAI and 37.1% for BRI. These proportions were substantially higher than those observed in the non-CKD group, where Q4 values represented only 23.4% of VAI and 23.4% of BRI measurements. Notably, among those with renal impairment, both VAI and BRI exhibited a graded increase in the proportion of Q4 values across quartiles [VAI: 11.4% (Q1) to 36.2% (Q4); BRI: 11.4% (Q1) to 37.1% (Q4)]. This graded pattern was absent in the non-CKD group, where the distributions of VAI (23.4–26.1%) and BRI (23.4–26.5%) remained markedly stable across all quartiles, with no obvious upward or downward trend.

Results of multivariable regression analysis (Table 3, Model3) demonstrated that higher levels of both VAI and BRI significantly associated with an increased risk of CKD, even after full adjustment for demographic and clinical covariates. When compared to the lowest quartile (Q1, reference group), the prevalence ratios for CKD showed a progressive up trend across successive quartiles of VAI (Q2: PR 1.08, 95% CI: 0.89–1.31; Q3: PR 1.28, 95% CI: 1.05–1.56; Q4: PR1.61, 95% CI: 1.32–1.98; *P* for trend < 0.001). Similarly, BRI exhibited a comparable graded association with CKD risk (Q2: PR 1.06, 95% CI 0.87–1.29; Q3: PR 1.09, 95% CI: 0.91–1.32; Q4: PR 1.26, 95% CI: 1.05–1.52; *P* for trend = 0.004).

**Table 3:** Logistic regression analysis of the association between VAI/BRI, and CKD.

Variables	Model 1		Model 2		Model 3	
	PR (95%CI)	*P*	PR (95%CI)	*P*	PR (95%CI)	*P*
VAI						
Continuous	1.01 (1.01, 1.02)	<0.001	1.01 (1.01, 1.02)	<0.001	1.01 (1.01, 1.01)	<0.001
Q1	Reference		Reference		Reference	
Q2	1.20 (1.00, 1.44)	0.049	1.23 (1.03, 1.47)	0.020	1.08 (0.89, 1.31)	0.445
Q3	1.49 (1.26, 1.77)	<0.001	1.57 (1.33, 1.86)	< 0.001	1.28 (1.05, 1.56)	0.015
Q4	2.09 (1.78, 2.45)	<0.001	2.20 (1.88, 2.58)	< 0.001	1.61 (1.32, 1.98)	< 0.001
*P* for trend		<0.001		<0.001		<0.001
BRI						
Continuous	1.24 (1.20, 1.29)	<0.001	1.19 (1.14, 1.24)	<0.001	1.08 (1.03, 1.13)	<0.001
Q1	Reference		Reference		Reference	
Q2	1.36 (1.13, 1.64)	<0.001	1.25 (1.04, 1.49)	0.02	1.06 (0.87, 1.29)	0.563
Q3	1.67 (1.40, 1.99)	<0.001	1.41 (1.19, 1.68)	<0.001	1.09 (0.91, 1.32)	0.353
Q4	2.29 (1.94, 2.70)	<0.001	1.84 (1.56, 2.17)	<0.001	1.26 (1.05, 1.52)	0.013
*P* for trend		<0.001		<0.001		0.004

Both VAI and BRI were divided into quartiles (Q1–Q4), Model 1: Unadjusted. Model 2: Adjusted for demographic variables (age, sex, ethnicity, education level, marital status, alcohol drinking, smoking, habitual salty diet, and physical exercise). Model 3: Additionally adjusted for hypertension, diabetes, hyperuricemia, and dyslipidemia based on Model 2. PR, prevalence ratio; CI, confidence interval.

### 3.4 Subgroup analysis

**Figure 3:**
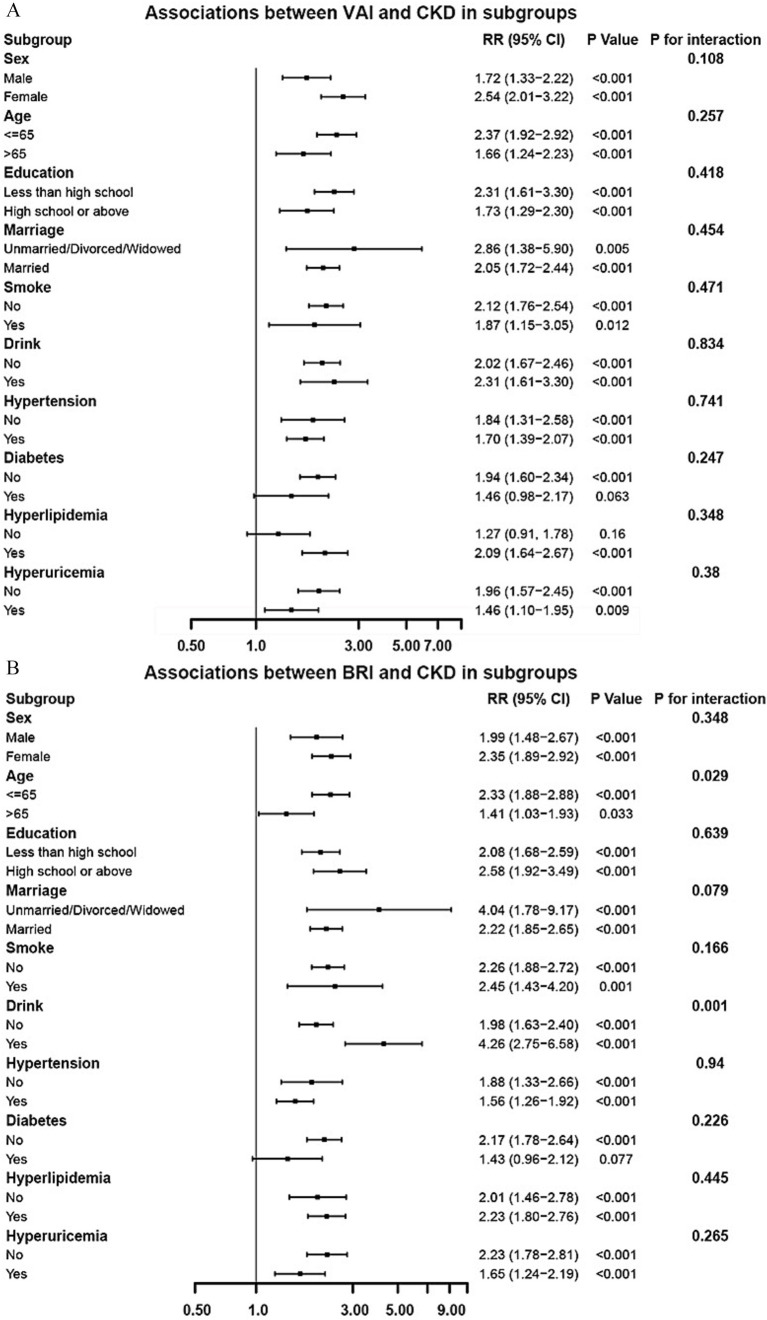
Subgroup analysis of the associations between VAI **(A)**/BRI **(B)** and CKD (adjusted based on Model 3 in Table 3).

Subgroup analyses were performed to evaluate the consistency of the associations between adiposity indices and CKD risk across different population strata (Figure 3). For VAI (Figure 3A), the positive association with CKD risk remained consistent across all predefined subgroups, including sex, age, education level, marital status, lifestyle factors, and comorbidities. No significant effect modification was observed in any of these subgroup (all *P* for interaction > 0.05), which underscores the robustness of the VAI-CKD association across diverse population characteristics. For BRI (Figure 3B), significant interaction effects were observed specifically with age and drink (both *P* for interaction < 0.05), whereas no statistically significant effect modification was observed in other subgroups.

### 3.5 Restricted cubic spline analysis and mediation analyses

**Figure 4:**
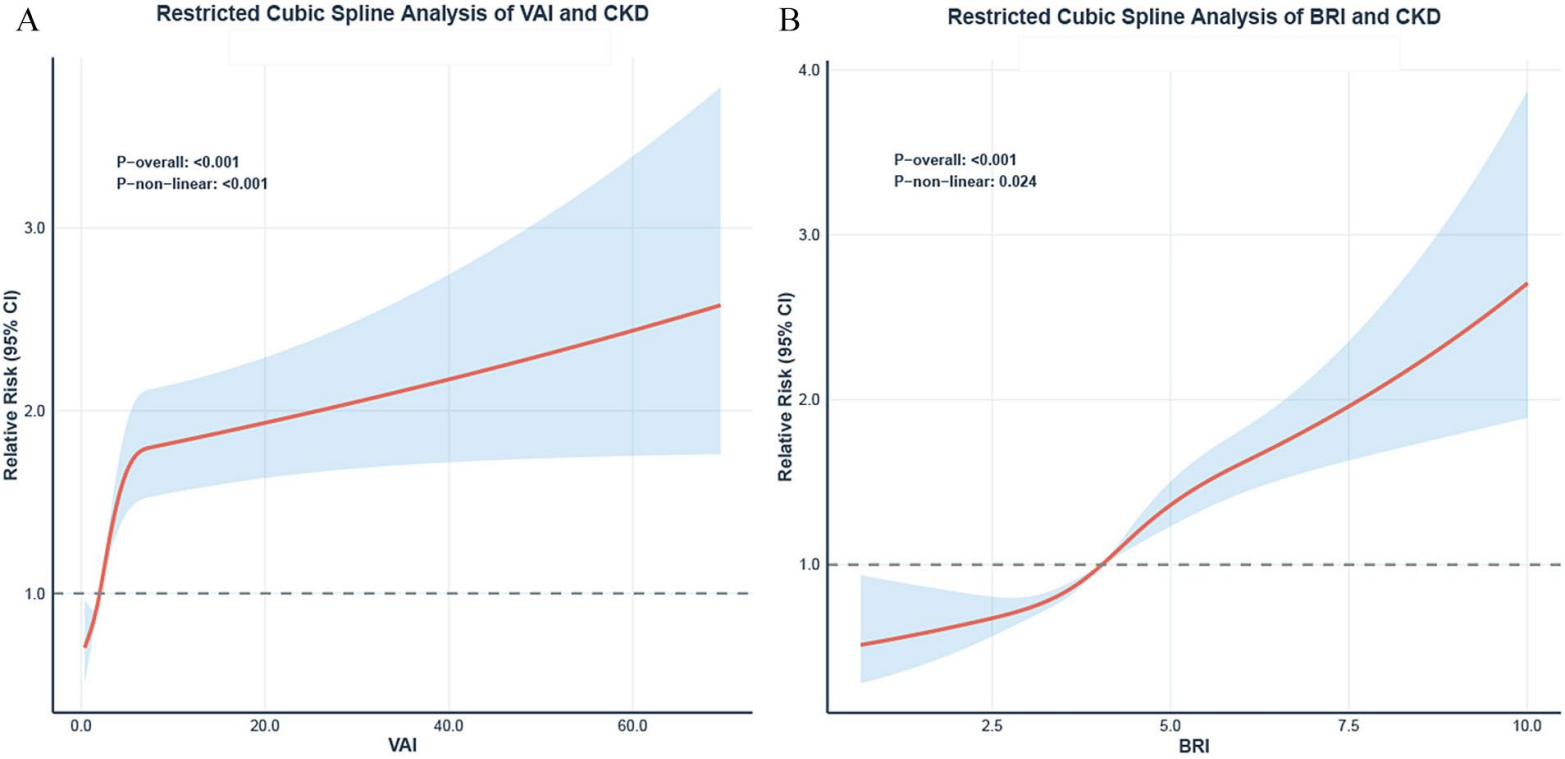
Restricted cubic spline analysis of VAI/BRI and CKD. Dose–response relationships between VAI **(A)**, BRI **(B)** and the risk of CKD. The solid lines represent the adjusted prevalence ratios (PR), and the shaded areas represent the 95% confidence intervals. All models were adjusted based on model 3 in Table 3. The reference point (PR = 1) was set at the 10th percentile of VAI and BRI. *P* for non-linearity is indicated.

Restricted cubic spline analysis (RCS) demonstrated significant nonlinear relationships between both VAI (Figure 4A) and BRI (Figure 4B) and the risk of CKD risk (both *P* for non-linearity < 0.05).

**Figure 5:**
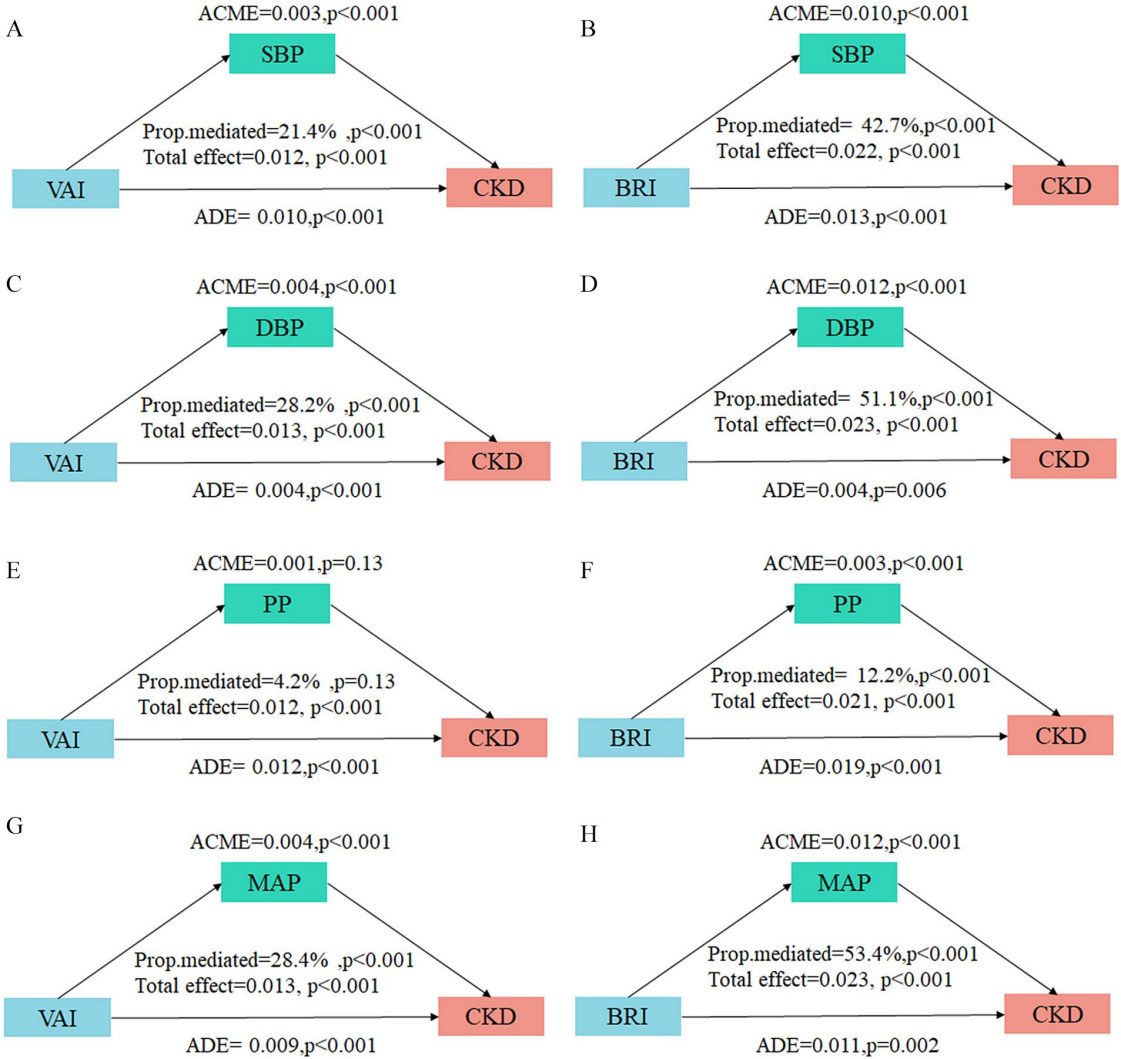
Mediation analysis of blood pressure indicators on the associations between VAI/BRI and CKD. The mediation analysis results display the decomposition of effects of blood pressure indicators on the associations between VAI/BRI and CKD. **(A,B)** SBP with VAI and BRI; **(C,D)** DBP with VAI and BRI; **(E,F)** PP with VAI and BRI; **(G,H)** MAP with VAI and BRI. ACME, average causal mediation effect; ADE, average direct effect. The proportion mediated. Adjusted for age, sex, ethnicity, education level, marital status, alcohol drinking, smoking, habitual salty diet, physical exercise, diabetes, hyperuricemia, and dyslipidemia.

To further investigate the potential mediating effect of blood pressure indicators in the relationship between adiposity indices and CKD, we conducted mediation analyses. The results demonstrated substantial mediation effects of SBP, DBP, and MAP for both the VAI-CKD and BRI-CKD pathways (Figure 5). Notably, the mediation proportions were consistently stronger for BRI compared to VAI across all significant blood pressure-mediated pathways. Specifically, the mediation proportions of blood pressure indicators in the VAI-CKD relationship were 21.4% (95% CI: 11.33–37.18) for SBP, 28.15% (95% CI: 14.92–43.84) for DBP, and 28.41% (95% CI: 16.10–48.81) for MAP. In contrast, the mediation effects were substantially more prominent for BRI, with proportions of 42.69% (95% CI: 30.94–66.40) for SBP, 51.16% (95% CI: 38.07–74.83) for DBP, and 53.38% (95% CI: 39.90–78.23) for MAP. Furthermore, PP demonstrated a significant mediation effect only in the BRI-CKD pathway (12.21, 95% CI: 7.28–20.70), while showing no significant mediating effect in the VAI-CKD pathway (4.23, 95% CI: −1.18–11.15).

## 4 Discussion

This cross-sectional study investigated the prevalence of CKD among adult population in Bobai County, Guangxi. The results revealed the prevalence of CKD in this population was 12.62% (95% CI: 11.94–13.33%), exceeding the 8.2% prevalence reported by Wang et al. (23) in the general Chinese adult population. This discrepancy may be attributed to regional differences in lifestyle (e.g., dietary patterns, physical activity levels), socioeconomic factors, or the high prevalence of metabolic comorbidities (e.g., obesity, hypertension) in the study population, underscoring the urgent need to identifying high-risk subgroups and implement precision prevention strategies for CKD in this region.

Within this epidemiological context, we identified significant positive associations between both VAI and BRI with CKD risk, demonstrating clear dose–response relationships. These findings are consistent with the growing body of evidence linking visceral adiposity to renal injury. For instance, a large retrospective cohort study confirmed that various visceral adiposity indices, including VAI and BRI, were significantly associated with both CKD risk and rapid kidney function decline (24). Zheng et al. (13) reported that higher VAI quartiles were associated with progressively elevated CKD: compared to the lowest quartile (reference), the relative risks for Q2, Q3, and Q4 were 2.24 (95%Cl: 1.70–2.95), 2.36 (95%Cl: 1.54–3.61), and 2.57 (95%Cl: 1.57–4.22), respectively. Furthermore, Fei et al. (24) demonstrated that each unit increase in BRI was associated with 10.5% higher odds of CKD (OR: 1.109; 95% CI: 1.040–1.183).

Collectively, these data support the notion visceral fat accumulation is a critical driver in CKD development and progression. For instance, visceral obesity has been established as a key risk factor for both CKD development and progression to end-stage renal disease (ESRD) (4). Notably, Peng et al. (25) innovatively proposed that the central obesity index Metabolic Score for Visceral Fat (METS-VF) is an independent risk factor for CKD, with CKD prevalence increasing significantly across METS-VF tertiles. On the contrary, lifestyle interventions targeting visceral obesity, such as dietary modification and increased physical activity, have been shown to slow CKD progression (26, 27). Our study extends these findings by validating the utility of VAI and BRI in southern Chinese adults, offering localized evidence to guide population-specific kidney disease prevention protocols.

The glomerulus is the primary early site of injury in obesity-related kidney disease, where mechanisms including insulin resistance, chronic inflammation, lipotoxicity, and hemodynamic alterations (28, 29). These mechanisms induce intraglomerular hypertension and hyperfiltration, leading to direct podocyte injury and disruption of the filtration barrier (30). This results in increased glomerular permeability, which is clinically detected as albuminuria (elevated UACR). A pivotal renal biopsy study of 95 severely obese patients with normal renal function undergoing bariatric surgery substantiates this mechanism, revealing characteristic early lesions (glomerulomegaly, podocyte hypertrophy, and mesangial cell proliferation) (31). During this stage, nephron compensation maintains normal eGFR, whereas UACR can sensitively detect such subclinical renal injury. In contrast, from a diagnostic standpoint, a marked decline in eGFR typically indicates more advanced structural damage (extensive glomerulosclerosis and interstitial fibrosis). However, that in a minority of conditions where the primary pathology targets the renal tubulointerstitium, a decline in eGFR can be the initial and predominant clinical manifestation, independent of significant albuminuria. Consistent with this pathological framework, our results demonstrated a more pronounced association between VAI/BRI and CKD defined by UACR≥30. Taken together, increased glomerular permeability is an early, pivotal event in obesity-related nephropathy that precedes and is partly independent of later GFR decline. This highlights the importance of prioritizing UACR screening in obese populations for the early intervention of CKD.

Among these, hypertension as a key manifestation of hemodynamic dysfunction, serves as a critical mediator linking visceral adiposity to renal injury. Experimental studies confirm that early-stage intra-glomerular hypertension and hyperfiltration are observable in obese animal models (32), validating the central role of blood pressure mechanisms. Our mediation analysis provides critical quantitative evidence and novel mechanistic insights into the blood pressure pathway. Our findings not only confirm the crucial mediating role of blood pressure indicators in the pathways connecting VAI/BRI to CKD, but also reveal differences between distinct adiposity indices.

For BRI (a purely morphological index reflecting trunk fat accumulation), the mediation proportions of blood pressure were substantially higher: 42.7% (SBP), 51.2% (DBP), and 53.4% (MAP). BRI’s focus on trunk fat likely explains this: trunk fat accumulation directly impacts blood pressure via mechanical compression (e.g., perirenal fat compression) and neuroendocrine activation (e.g., SNS/RAAS stimulation), making MAP (a marker of renal perfusion pressure) the strongest mediator (53.4%). Research has demonstrated that a high BRI is closely associated with hypertension (18). On one hand, substantial visceral and perirenal fat accumulation directly compresses renal parenchyma, increasing renal pressure and inducing medullary hypoxia (33, 34). In the Framingham Heart Study, individuals with high perirenal fat (“fatty kidney”) demonstrated over a two-fold higher risk of hypertension (35). Concurrently, increased intra-abdominal pressure further alters renal hemodynamics and promotes sodium retention (36). On the other hand, adipose tissue, particularly visceral fat, autonomously produces angiotensinogen (37), triggering RAAS overactivation. Increased angiotensin II levels induce vasoconstriction and promote sodium and water retention, raising blood pressure. Experimental models substantiate this pathway: fasting suppresses angiotensinogen expression in rat VAT, while refeeding markedly upregulates its production and elevates blood pressure (38). Similarly, mice with VAT-specific angiotensinogen overexpressing develop hypertension (39). Furthermore, adipocyte-derived mediators such as leptin act on the hypothalamus to induce SNS overactivation, increasing renal vascular resistance and enhancing sodium reabsorption (40). Notably, leptin neutralization or inhibition of hypothalamic leptin receptors normalizes blood pressure in obese rodents. Conversely, leptin-deficient mice and humans with leptin or leptin receptor mutations exhibit lower blood pressure despite severe obesity (41, 42).

In contrast, VAI is a functional composite measure that focuses more on assessing the metabolic activity of VAT. Its relatively lower mediation proportion in the blood pressure pathway (21.4% for SBP, 28.2% for DBP, 28.4% for MAP) suggests that VAI-associated renal injury primarily occurs via non-hypertensive pathways, including more pronounced insulin resistance, direct lipotoxic effects, and chronic inflammatory states. Elevated levels of insulin resistance surrogates (e.g., TYG) are significantly correlated with a higher probability of uncontrolled, resistant, and refractory hypertension and with increased disease severity (43). Additionally, adipose tissue dysfunction induces chronic low-grade inflammation and oxidative stress impairing vascular endothelial function and reducing nitric oxide bioavailability, disrupting blood pressure homeostasis. Several studies have indicated that VAI is more strongly associated with cardiovascular risk and MetS than other anthropometric indices (e.g., WC, BMI, BRI, and WHR) (44–46). Notably, PP (a marker of arterial stiffness) exhibited a significant mediation effect only in the BRI-CKD pathway (12.2%), indicating that BRI-defined central obesity may be closely linked to arteriosclerosis. This association may involve multiple signaling pathways, such as integrin-mediated signal transduction, promoting phenotypic switching of vascular smooth muscle cells phenotypic switching and extracellular matrix remodeling, ultimately leading to vascular wall thickening and reduced elasticity (47).

The observed differences indicate that BRI and VAI may reflect obesity-associated health risks from distinct dimensions. This understanding provides a basis for selecting appropriate risk assessment tools in different practical scenarios. Notably, BRI requires only height and waist circumference, enabling rapid, low-cost, and highly reproducible assessment without laboratory tests. This makes BRI a particularly cost-effective tool for early CKD risk identification in resource-limited or large-scale screening settings (e.g., primary care, epidemiological surveys). In contrast, VAI incorporates lipid parameters, providing additional information on metabolic status. In clinical settings where blood testing is available, it can be used for more comprehensive metabolic risk assessment in high-risk individuals. The two measures are complementary and can serve different stages of prevention and management.

This study has several limitations. First, due to the cross-sectional observational study design, the causal relationships between VAI/BRI, and CKD cannot be fully determined, prospective cohort studies are needed to confirm temporal associations. Second, although multiple confounding factors were adjusted for in our analyses, the potential influence of residual confounding cannot be entirely excluded. Finally, the study did not extensively explore other potential mediators (e.g., inflammatory cytokines, insulin levels, or lipotoxicity-related biomarkers), which may contribute to obesity-related kidney injury. Future research should involve prospective cohort studies or Mendelian randomization analyses to conduct comprehensive multi-mediator analyses, elucidating the full mechanistic spectrum.

## 5 Conclusion

In conclusion, our study demonstrates that the novel adiposity indices VAI and BRI are independently associated with CKD risk in southern Chinese adults, even after adjusting for potential confounders. Moreover, mediation analysis further confirms that blood pressure plays a significant mediating role in these associations—with stronger effects observed for BRI than VAI. These findings establish a foundation for the development of targeted CKD prevention and management strategies, can identify high-risk individuals, while blood pressure control may mitigate CKD risk in those with elevated adiposity. Ultimately, this work provides valuable tools to address the growing global burden of CKD.

## Data Availability

The raw data supporting the conclusions of this article will be made available by the authors upon reasonable request.

## Glossary

ALB: Urinary Albumin
BMI: Body Mass Index
BRI: Body Roundness Index
BUN: Blood Urea Nitrogen
CKD: Chronic Kidney Disease
CVD: Cardiovascular Disease
DBP: Diastolic Blood Pressure
FPG: Fasting Plasma Glucose
eGFR: Estimated Glomerular Filtration Rate
HDL-C: High-Density Lipoprotein Cholesterol
HKB: Hakka Biobank
LDL-C: Low-Density Lipoprotein Cholesterol
MAP: Mean Arterial Pressure
METS-VF: Metabolic Score for Visceral Fat
PP: Pulse Pressure
PR: Prevalence Ratio
RAAS: Renin-Angiotensin-Aldosterone System
RCS: Restricted Cubic Spline
SBP: Systolic Blood Pressure
Scr: Serum Creatinine
SNS: Sympathetic Nervous System
TC: Total Cholesterol
TG: Triglycerides
UCR: Urinary Creatinine
UA: Uric Acid
UACR: Urinary Albumin-to-Creatinine Ratio
VAI: Visceral Adiposity Index
VAT: Visceral Adipose Tissue
WC: Waist Circumference
